# From the Ocean to the Operating Room: The Role of Fish Skin Grafts in Burn Management—A Systematic Review

**DOI:** 10.3390/jcm14165750

**Published:** 2025-08-14

**Authors:** Mohamed Marzouk El Araby, Gianluca Marcaccini, Pietro Susini, Francesco Ruben Giardino, Mirco Pozzi, Vera Pizzo, Luca Grimaldi, Alessandro Innocenti, Roberto Cuomo, Giuseppe Nisi, Cristian Pascone, Antonio Di Lonardo

**Affiliations:** 1 Surgery and Neuroscience—Plastic Surgery Unit, Department of Medicine, University of Siena, Policlinico Santa Maria “Le Scotte”, 53100 Siena, Italy; m.marzouk@outlook.it (M.M.E.A.); gianlu32@gmail.com (G.M.); mircovirgilio.pozzi@gmail.com (M.P.); vera29@hotmail.it (V.P.); luca.grimaldi@unisi.it (L.G.); robertocuomo@outlook.com (R.C.); giuseppe.nisi@unisi.it (G.N.); 2 Plastic Surgery Unit, Mater Olbia Hospital, SS 125 Orientale Sarda, 07026 Olbia, Italy; f.ruben.giardino@gmail.com; 3 Plastic and Reconstructive Microsurgery, Careggi University Hospital, Largo Giovanni Alessandro Brambilla, 3, 50134 Florence, Italy; a.innocenti@unifi.it; 4 Burn Unit, Ospedale Cisanello, Via Paradisa, 2, 56124 Pisa, Italy; christianpascone@hotmail.it (C.P.); a.dilonardo@ao-pisa.toscana.it (A.D.L.)

**Keywords:** fish skin graft, acellular fish skin, burn wound management, xenograft, biological skin substitute, thermal injury, novel burn treatments, dermal regeneration, wound coverage, resource-limited settings, scaffolds, omega 3

## Abstract

**Background**: The treatment of burns is a socio-economic challenge for both patients and the National Health Service. Early debridement and skin graft reduces the risk of local and systemic complications. However, when skin autografting is unfeasible or contraindicated, alternative options are required. Recent research has introduced new potential tools: fish skin grafts (FSGs). This systematic review focuses on FSGs with the aim of improving the management of burn patients. **Methods**: A systematic search on articles concerning FSG for the treatment of burns was performed by searching PubMed, Web of Science and Embase according to the PRISMA statement. Clinical trials, retrospective studies, case series and case reports were included. **Results**: A total of 36 studies were identified through the search strategy and imported for screening. After duplicate removal, 26 studies were considered. Based on predetermined criteria, 20 full texts were assessed for eligibility, leaving 18 articles to be included in the systematic review. **Conclusions**: By virtue of the safety and effectiveness of FSGs, including low risk of zoonosis transmission and valuable outcomes even in austere environments, FSGs could represent a new alternative for the treatment of burns.

## 1. Introduction

Over 180,000 burn deaths are reported annually according to the World Health Organization, leading to a significant socio-economic impact [1]. Developed countries, despite a global decline in incidence and mortality, have shown an increase in burn injuries, especially in the military context [2]. This rise could be attributed to changes in combat methods, including aggressive drones and long-range attacks [3].

The current mainstay of therapy for deep partial-thickness burns (DPTBs) and full-thickness burns (FTBs) is represented by early debridement and skin autograft. The procedure has been demonstrated to reduce local and systemic complications, hospital times, infections and deaths [4,5,6]. However, it is inherently limited by donor site morbidity, particularly in patients with extended burns [7].

Therapeutic alternatives include allogenic grafts and acellular dermal matrices (ADMs) of porcine, bovine and ovine origin. These scaffolds promote cell proliferation and skin re-epithelialization in a wide context of wounds including ulcers, burns and split-thickness skin graft (STSG) donor sites. However, allogeneic grafts and ADMs are not free from limitations, considering the potential risks of autoimmune response and rejection, infection and zoonotic transmission [8,9]. To minimize these risks, ADM processing and the viral inactivation process are accomplished by specific detergents capable of removing various soluble components. Detergents are highly effective, but also remove lipids, glycans, elastin, hyaluronic acid and other biocomponents. These have been demonstrated to play a relevant role in the healing process [10]. In addition, there is a religious obstacle associated with porcine ADM such that it cannot be applied to all patients [11,12].

**Figure 1:**
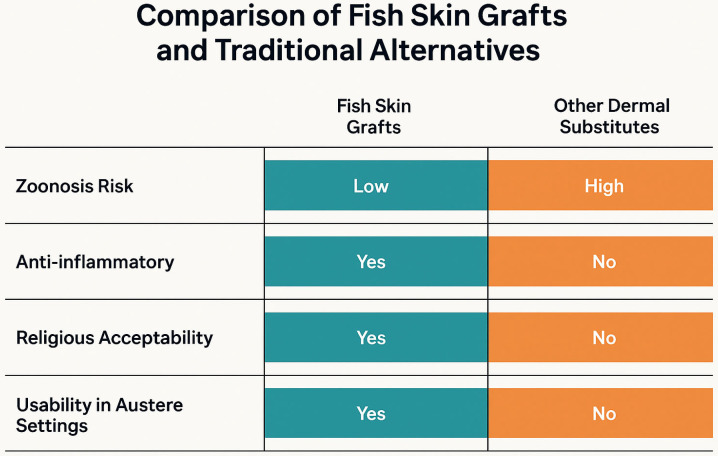
Summary of benefits of FSGs in relation to traditional dermal matrices.

Recent research has introduced a new type of xenograft: fish skin grafts (FSGs). FSGs are safe products with no documented risk of zoonotic transmission (Figure 1) [13,14,15,16,17]. A less aggressive decellularization process is required, allowing for preservation of biocomponents like proteoglycans, glycoproteins, soluble collagen, elastin, lanolin, fibronectin and omega-3 polyunsaturated fatty acids (PUFAs) [13,14,15,16,17]. Notably, the unique presence of PUFAs has shown antibacterial, antiviral and anti-inflammatory activities through inhibition of macrophage secretion of the proinflammatory interleukin 1-beta [14,18,19,20,21]. PUFAs also reduce pain by down-regulating nociceptive pathways [14,18,19,20]. Four FSG species are available: North Atlantic cod (*Gadus morhua*), Nile tilapia (*Oreochromis niloticus*), silver carp (*Hypophthalmichthys molitrix*) and grass carp (*Ctenopharyngodon idellus*). This manuscript reports a systematic review of the literature on FSGs with the aim of improving the management of burn patients.

## 2. Materials and Methods

### 2.1. Data Sources and Search Strategy

Following the PRISMA guidelines, we performed a systematic search of PubMed, Embase and Web of Science from database inception through 1 February 2024. Controlled-vocabulary headings (MeSH/Emtree) and free-text keywords for FSGs (“fish skin graft*,” “acellular fish skin”, “Omega3 wound matrix”, “xenograft”, “North Atlantic cod”, “Gadus morhua”, “Nile tilapia”, “Grass carp”, “Silver carp”, “Kerecis”) were combined with burn-related terms (“burn*”, “thermal injury*”, “scald*”) using Boolean operators. Searches were limited to English-language publications and reference lists of all included articles and relevant reviews were hand-searched to identify additional studies.

### 2.2. Study Selection

We included original research articles reporting on FSGs for burn management—specifically, preclinical trials, randomized and non-randomized clinical trials, retrospective cohort studies, case series and case reports—and excluded reviews, editorials, commentaries, conference abstracts without full text, protocols, non-English publications and studies not focused on burn injuries. Two reviewers independently screened titles and abstracts, then assessed full texts for eligibility in a two-step process; disagreements were resolved by consensus.

### 2.3. Data Extraction

Data were extracted independently by two authors into a standardized spreadsheet. Extracted variables included: author, year, country, study design, sample size, subject model (human or animal), burn etiology and depth, fish species and graft processing method, application protocol, primary and secondary outcomes (time to re-epithelialization, graft take rate, pain scores, number of dressings, adverse events) and follow-up duration. Discrepancies were resolved through discussion. The collected articles were brought to the attention of the senior author (AdL) for final approval.

### 2.4. Data Synthesis

Owing to clinical and methodological heterogeneity among the included studies, we did not perform a meta-analysis. Instead, we synthesized findings narratively and summarized key study characteristics and outcomes in tabular form. No formal risk-of-bias assessment was conducted.

## 3. Results

**Figure 2:**
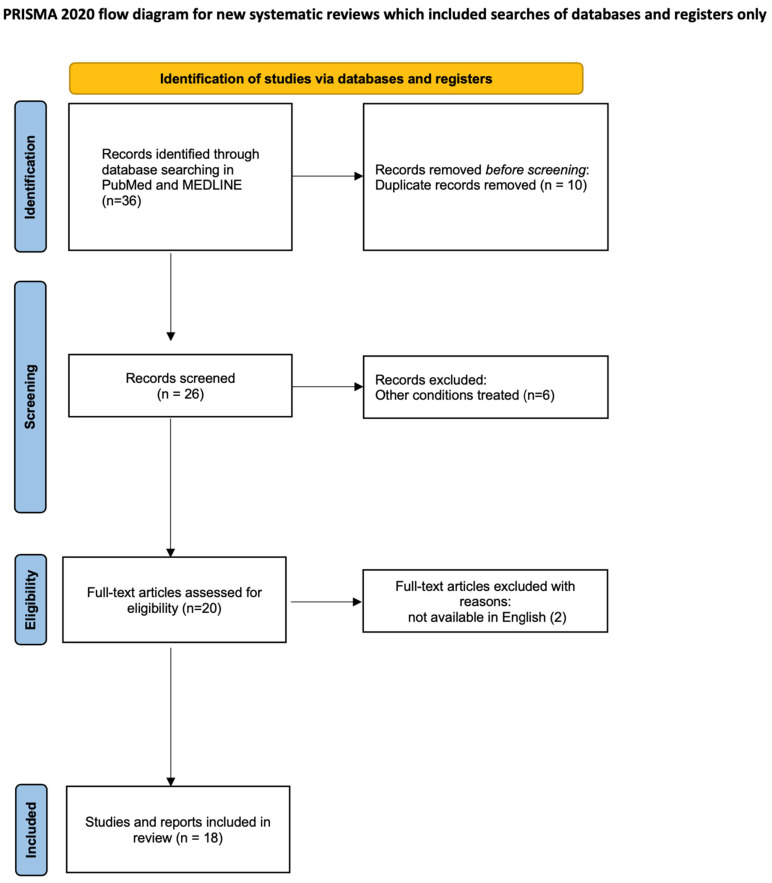
PRISMA diagram.

A total of 36 studies were identified and imported for screening. After duplicate removal and application of predefined eligibility criteria, 18 articles were ultimately included in the systematic review (Figure 2). These comprised comparative and retrospective preclinical studies, randomized and non-randomized clinical trials, case series and case reports, encompassing both human and veterinary models. The main reported outcomes included re-epithelialization time, pain levels, number of dressing changes and overall graft performance.

### 3.1. Comparative Preclinical Studies

Wei et al. [22] evaluated wound healing in DPTBs induced in Kunming mice. Three healthy male mice received second-degree burns on their backs and were randomly assigned to one of three groups: no treatment, a commercial product control or an acellular FSG derived from silver carp. Wounds were assessed at days 5, 8, 15 and 20. By day 14, most lesions were healed, but the silver carp group achieved a mean wound healing rate of 93.89% ± 3.15%, exceeding the no-treatment and commercial groups by 5.47% and 7.26%, respectively. In addition, porosity was 79.64% ± 0.17%, tensile strength 4.36 ± 0.06 and cell proliferation rate 117.79% ± 15.26%, indicating scaffold performance comparable to the commercial product.

Varon et al. [23] compared five treatments in five pigs with twelve DPTBs each, created at 100 °C for 15 s. One hour after burn induction, wounds were debrided with sterile saline and gauze. Six burns per animal received either 1% silver sulfadiazine (SSD) or one of four experimental therapies: irradiated sterile human skin allograft (IHS), biodegradable temporizing matrix (BTM), polylactic acid skin substitute, hyaluronic acid ester matrix (HAM) or decellularized FSG from North Atlantic cod. At day 28, wound contraction with FSG was 26.5% ± 8.41%, second only to IHS (28.00% ± 6.40%), with no significant difference versus SSD. Revascularization was high with IHS (133.62 ± 5.32), HAM (129.44 ± 7.94) and FSG (120.35 ± 2.13). Mean scar elevation index exceeded 20 for all treatments except FSG (16.5 ± 2.91). Complete re-epithelialization was achieved by day 28 with FSG, IHS and HAM. The rate of burn depth progression was slower in wounds treated with FSG.

Stone et al. [24] conducted a randomized, double-blind trial in Yorkshire pigs comparing North Atlantic cod grafts with fetal bovine dermis (FBD). Twenty-four 5 × 5 cm burns were created and then excised 24 h later. FSG or FBD were applied and reapplied as needed. Wounds were assessed at days 7, 14, 21, 28, 45 and 60. At day 14, re-epithelialization in the FSG group was 50.2% compared with 23.5% in the FBD group. Reduction in original wound size at day 14 was 93.1% for FSG versus 106.7% for FBD. No differences in transepidermal water loss were reported. Hydration measurements were lower for FSG at day 21. Laser speckle analysis showed a 4.9-fold increase in blood flow for FSG versus 3.1-fold for FBD. Both grafts induced granulation tissue formation, but FSG did so 7 days earlier, promoting faster re-epithelialization and greater wound contraction.

Shi et al. [25] treated five New Zealand White rabbits with five burns per side (twenty wounds total). Five wounds were dressed with gauze, five with gauze and petrolatum, five with grass carp-derived FSG and five with porcine skin collagen (PSC). Lesions were evaluated at days 0, 7, 14, 21 and 28 for water uptake, water vapor transmission rate and wound area. FSGs absorbed 47.8 times their weight in water versus 27.4 times for PSC, and both scaffolds exhibited adequate transmission rates. By day 3, the wound area increased by 20% with gauze, 15% with FSG and PSC and 10% with petrolatum. At day 9, wounds treated with FSG and PSC showed greater reduction in area than petrolatum. By day 28, PSC and FSG groups achieved complete healing, while the other groups remained partially unhealed. Overall, the higher water uptake, slower loss and favorable transmission rates suggest FSG as a viable alternative to PSC.

### 3.2. Retrospective Preclinical Study

Mauer et al. [26] retrospectively reviewed seventeen animals (thirteen dogs, four cats) treated across thirty-one veterinary hospitals in 2022. Wounds of various etiologies—including five burns—were managed with North Atlantic cod-derived FSG. Second-intention healing was achieved between 26 and 145 days (median 71 days) without adverse events attributed to the graft.

### 3.3. Clinical Studies

Yoon et al. [27] enrolled fifty-two burn patients requiring skin graft donor site coverage. Split-thickness graft donor sites were treated by Kerecis™ (Kerecis, Ísafjörður, Iceland) FSG alone or in combination with ProHeal™ bovine collagen (MedSkin Solutions Dr. Suwelack AG, Billerbeck, Germany). Healing rate was assessed by ImageJ analysis software, version 1.53t (National Institutes of Health, Bethesda, MD, USA), defining healing as >95% coverage. Kerecis™ reduced the mean healing time from 11.9 ± 1.4 days to 9.1 ± 1.0 days without treatment, and from 13.1 ± 1.4 days to 10.7 ± 1.5 days versus ProHeal™.

Lima et al. [28] conducted a phase III trial in 2021 with 115 patients having superficial partial-thickness burns (SPTBs) on ≤15% of total body surface area (TBSA). Patients were randomized to Nile tilapia graft or 1% SSD. After wound debridement, SSD was applied to 58 patients and FSG to 57. FSG reduced the mean re-epithelialization time (9.7 ± 0.6 days vs. 10.2 ± 0.9 days), number of dressing changes (1.6 ± 0.7 vs. 4.9 ± 0.5) and saved an average of USD 8 per patient. Pain on VAS was lower with FSG (20.5 ± 8.4 vs. 29.2 ± 13.1) and von Frey testing (332.6 ± 163.3 g vs. 483.5 ± 312.0 g). Analgesic dipyrone use was half that of SSD, with no difference in tramadol.

Lima et al. [29] compared freeze-dried Nile tilapia FSG with a silver-impregnated carboxymethylcellulose dressing in 24 patients with burns on ≤10% TBSA. Evaluations at treatment, day 5 and day 10–11 showed fewer dressing changes with Nile tilapia FSG (median 1 vs. 2) and reduced VAS scores after medication (13.96 ± 8.76 vs. 24.79 ± 11.05). No significant differences were found in von Frey pain testing, analgesic intake or anxiety.

Lima et al. [30] performed a phase 2 trial in 62 patients divided into three groups by burn depth and TBSA. After water and 2% chlorhexidine debridement, patients received either Nile tilapia FSG or 1% SSD. In group A (<10% SPTB), the FSG group achieved a 1.43-day gain in re-epithelialization; in group B (10–20% SPTB) 1.14 days; and in group C (5–15% DPTB) 3.20 days. Pain reduction was significant in groups B and C, but not in A. Dressing changes were fewer with FSG in all groups. Dipyrone use was reduced in group C and ketamine in group B; group C also showed reduced fentanyl use.

Lima et al. [31] conducted a pilot study in 30 pediatric SPTBs (<20% TBSA). FSG reduced time to >95% re-epithelialization by 0.40 days (10.07 ± 0.46 vs. 10.47 ± 0.74), and decreased dressing numbers and ketamine use. Pain scores did not differ. Similar healing times were reported in other reports focused on free flaps donor-sited [32].

### 3.4. Case Series and Case Reports

Reda et al. [3] described three patients with DPTBs after blast injuries. FSG promoted rapid granulation tissue formation, facilitating earlier grafting and reducing flap requirements. Dawson et al. [33] reported a spayed American Bulldog with mixed SPTB/FTB, TBSA > 50%, treated with FSG and negative-pressure therapy. Re-epithelialization reached 30% at day 18 and 50% at day 35. Wallner et al. [34] compared enzymatic debridement plus FSG with Suprathel™ (PolyMedics Innovations GmbH, Denkendorf, Germany) and split-thickness grafts in 12 patients. FSG achieved re-epithelialization 12.7 days earlier than grafts and 23 days earlier than Suprathel™. Superior scar quality metrics were reported. Lima et al. [35] and Costa et al. [36] presented two single-patient reports of SPTBs successfully treated with Nile tilapia FSG, achieving full re-epithelialization by day 10. Sandness et al. [37] treated a dog with DPTB using cod FSG, observing 70% length and 90% width reduction by day 15, up to 95% by day 28. Alam et al. [38] reported 10 SPTB patients treated with Kerecis™, achieving 100% re-epithelialization in 11.5 days (range 10–16) with no infections and mean VAS pain score of 2.3 (range 1–4) at day 7. Finally, Lima et al. [39] described a young adult with mixed burns treated with Nile tilapia FSG. The man achieved re-epithelialization without dressing changes or adverse events in 12 days for SPTB and 17 days for DPTB, respectively.

Key characteristics and results are summarized in Table 1 and Table 2.

## 4. Discussion

Early debridement and skin graft represents the cornerstone treatment for both DPTB and FTB. However, homologous and allogeneic skin could be poorly available in extended burns. Skin banks are also limited and sometimes difficult to find. Indeed, recent research has focused on alternative methods. The advent of fish-derived cell matrices could represent an important innovation. At the time of this review, four FSGs are currently available: silver carp (*Hypophthalmichthys molitrix*)-, North Atlantic cod (*Gadus morhua*)-, grass carp (*Ctenopharyngodon idellus*)- and Nile tilapia *(Oreochromis niloticus*)-derived FSGs.

Silver carp is native to eastern Asia and belongs to the *Cyprinidae* family. Unlike North Atlantic cod, it can be farmed, thereby ensuring strict traceability [22]. Its skin collagen exhibits a denaturation temperature of 29 °C, which is 11 to 14 °C higher than that of North Atlantic cod [40,41]. The porosity of the silver carp-derived FSG, which reflects in vitro cell colonization and in vivo tissue growth, was 79.64% ± 0.17% [22], higher than that of Kerecis™ (63.6% ± 6.4%) [25,26]. However, its tensile strength (4.36 ± 0.06 MPa) was lower than that of tilapia FSG (11.29 ± 2.27 MPa) and porcine ADM (11.76 ± 2.46 MPa) [42,43,44].

Grass carp, another member of the *Cyprinidae* family native to Central Asia, yields an acellular scaffold with a porosity of 98.1% ± 0.5% [25], the highest among all scaffolds herein described. Porosity is critical for wound healing. A porosity of 60 to 90% is considered optimal as it supports cellular response, oxygen and nutrient transport, and matrix component development [45]. However, increased porosity results in reduced mechanical strength, underscoring the need for balance between these properties [27].

Although humans and fish diverged over 350 million years ago, fish skin shares several structural similarities with mammalian skin [16]. It is rich in omega-3 PUFAs, eicosapentaenoic acid (EPA) and docosahexaenoic acid (DHA) [46]. These are responsible for documented antibacterial, anti-inflammatory [12,13,18,47,48,49,50,51,52,53], antiviral [19,51] and immunomodulatory activities [50,54,55,56].

Unsaturated fats, particularly PUFAs, are considered beneficial in the diet compared to saturated fats. Among PUFAs, fish skin, fish oil and some algae sources have been shown to contain omega-3 fatty acids [50,54,55,56]. Preliminary studies have shown that these fats can reduce cardiovascular risks and, in some cases, lower incidence of specific cancers. The most abundant omega-3 PUFA in cell membranes is DHA [50,54,55,56]. DHA plays a key role in brain and retinal function with potential associations with reduced risk of breast cancer. Overall, the role of PUFAs and omega-3 seems promising and further evidence is expected.

Long-chain omega-3 fatty acids (LCUFAs) are present in fish skin and respiratory secretions, where they exert antibacterial effects through mechanisms such as bacterial membrane disruption [57]. Several studies have shown that cystic fibrosis patients deficient in omega-3 and omega-6 fatty acids, particularly DHA, exhibit an excess of arachidonic acid [58]. In these patients, oral DHA supplementation corrects the imbalance and leads to clinical improvement [59,60].

In a recent study by Homes et al. [18], the authors tested the antibacterial activity of LCUFAs, particularly DHA, in vitro and in vivo using *Galleria mellonella* larvae infected by *Burkholderia cenocepacia* K56-2. Following the administration of 50 mM DHA, antibacterial effects were documented [18]. In another study, Magnusson et al. [13] evaluated cell ingrowth and antibacterial properties of North Atlantic cod-derived FSG by placing the xenograft between two chamber units, one containing bacteria and the other containing sterile media. They demonstrated that the matrix acted as a bacterial barrier for 48 to 72 h. The effect was further enhanced by supplemental omega-3 [13].

Analysis of available literature shows that no randomized clinical trial on FSG for FTB treatment is currently available. Given that lesion depth is a key factor prolonging hospital stay and increasing costs, and FTBs are associated with higher morbidity and greater need for surgical intervention, this constitutes a significant limitation. The lack of data in this subgroup limits the generalizability of our conclusions and highlights the need for additional clinical trials focused on FSG in deep burns. In a healthcare policy context where hospitalization costs must be minimized, it would be useful to evaluate cost savings associated with FSG compared to other treatments, as performed by Lima et al. [28], rather than focusing solely on healing time.

The wound healing process consists of four phases: hemostasis, inflammation, proliferation and remodeling [61,62,63]. The inflammatory phase is crucial, and its failure to resolve impedes healing [61]. Omega-3 fatty acids, DHA and EPA, promote resolution of inflammation through the formation of specialized pro-resolving lipid mediators (SPMs) [50,62,63]. SPMs, including resolvins, protectins and maresins, arrest inflammation by reducing lymphocyte infiltration, modulating antibacterial gene expression [50] and decreasing chemokine production [63].

**Figure 3:**
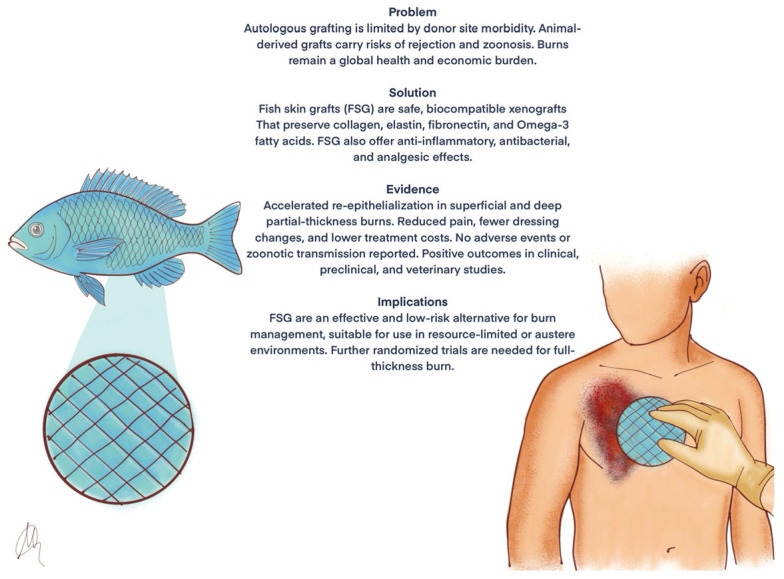
Overview of key concepts.

Given the significant impact of burns on healthcare systems in terms of social costs and hospitalization, healing time is a key factor in patient management. Analysis of studies reporting burn depth (Table 2) shows wide variability in terms of complete re-epithelialization times. This variability may be explained by multiple factors, including the fish species used for the xenograft, the type of dressing covering the graft and patients’ morbidities. Donor site grafts re-epithelialize in 9.1 ± 1 to 31.5 days. For SPTBs, re-epithelialization occurs in 9.7 ± 0.6 to 10.56 ± 1.13 days, for DPTBs it ranges from 14 to 28 days. Given that complete re-epithelialization of SPTBs typically occurs within two weeks and of DPTBs within three weeks [30], these data indicate that FSG can reduce healing time by several days (Figure 3).

### Study Limitations

The main limitation of this study is that it does not provide a treatment algorithm for burn wounds including a potential role for FSG. However, the paper offers a comprehensive assessment of FSG, exploring the topic from various perspectives, based on the best available evidence. Direct comparisons are limited by differences in burn depth, fish species used, FSG processing methods and outcomes. Data on pain reduction and cost-effectiveness are inconsistent. The narrative synthesis lacks critical appraisal of conflicting or inconsistent findings. While subgroup analyses were not feasible, future trials should carefully consider these factors to better identify which clinical scenarios could benefit the most from FSG application. Regarding the systematic review, the included studies present numerically variable samples and analyze different aspects of FSG and burn wounds. No statistical analysis was performed, and no formal risk of bias assessment was conducted due to the high heterogeneity of study designs and outcomes. Moreover, we did not apply a GRADE assessment for certainty of evidence, given the heterogeneity in study designs, populations and outcome measures, which precluded the use of standardized grading across studies. Future systematic reviews based on more homogeneous datasets and randomized clinical trials may allow for formal GRADE evaluation. Further investigations are warranted.

## 5. Conclusions

FSGs are simple to use and cost-effective innovative ADMs. Evidence from preclinical and clinical studies shows that FSG could represent a new potential alternative for the treatment of burns. The minimally invasive processing of the product allows for preservation of its structural characteristics, ensuring anti-inflammatory properties, while preventing the risk of zoonosis transmission. Particularly, the unique presence of PUFAs accounts for FSGs’ peculiar antibacterial, antiviral and anti-inflammatory properties. FSGs have been demonstrated as an effective treatment for SPTBs. Specifically, in selected clinical scenarios, FSGs reduce re-epithelialization time, minimize pain and lower the need for dressing changes compared to traditional treatments. Moreover, they have been employed in FTBs with promising outcomes. Despite the early evidence, further randomized clinical trials on the application of FSG for FTBs are expected. Overall, FSGs may be considered as a valuable adjunct in selected patients when conventional grafts are unavailable or contraindicated. Further research is warranted.

## Figures and Tables

**Table 1:** Summary of evidence.

Study (Author, Year)	Study Type	Burn Etiology and Depth	Fish Skin Type	Comparison Product	Cohort/Animal Model	Treatment Period	Endpoint(s)	Main Results
Wei et al., 2023 [22]	Comparative Animal Study	Flame, DPTB	Silver carp (*Hypophthalmichthys molitrix*)	Commercial product	3 Kunming mice (3 groups: no treatment, commercial product, fish skin)	20 days	Wound healing rate	Superior healing in the fish skin group (93.9%) compared to no treatment (+5.5%) and commercial (+7.3%).
Reda et al., 2023 [3]	Case Series	Combat injury, SPTB, DPTB	North Atlantic cod (*Gadus morhua*)	None	3 patients with burns and blast injuries	7 days	Granulation tissue formation	Rapid granulation tissue formation.
Dawson et al., 2022 [33]	Case Report	Flame, SPTB, DPTB	North Atlantic cod (*Gadus morhua*)	None	1 dog with SPTB and DPTB burns	35 days	Wound healing	More rapid healing.
Varon et al., 2023 [23]	Randomized Trial	Flame, DPTB	North Atlantic cod (*Gadus morhua*)	Human allograft, BTM, PLA, HAM	5 anesthetized pigs	28 days	Contraction, revascularization, re-epithelialization, scar index, colony-forming units (CFU)	100% re-epithelialization at day 28.
Yoon J. et al., 2022 [27]	Prospective Comparative Study	Flame, SPTB, DPTB	North Atlantic cod (*Gadus morhua*)	Bovine collagen (ProHeAL)	52 patients (STSG donor sites)	Up to 17 days	Healing time	Faster healing by ~2 days with FSG compared to no treatment and bovine collagen.
Mauer et al., 2022 [26]	Retrospective Animal Study	Flame, SPTB, DPTB	North Atlantic cod (*Gadus morhua*)	None	17 animals (13 dogs, 4 cats), 3 with burns	-	Wound closure	Time to wound closure: 26–145 days (median: 71).
Wallner et al., 2022 [34]	Retrospective Case–Control Study	Flame, DPTB	North Atlantic cod (*Gadus morhua*)	Synthetic skin (Suprathel^®^), STSG	12 patients	28 days	Re-epithelialization time, scar quality	Shorter re-epithelialization time with FSG (22 days) compared to STSG (12 days) and Suprathel (23 days).
Stone et al., 2021 [24]	Pre-clinical Trial	Flame, DPTB	North Atlantic cod (*Gadus morhua*)	Fetal bovine dermis (FBD)	6 Yorkshire pigs	28 days	Closure rate, transepidermal water loss, hydration, blood flow	Significantly faster re-epithelialization at day 14 compared to FBD (50.2% vs. 23.5%).
Lima Júnior et al., 2021 [28]	Phase III RCT	Flame, SPTB, DPTB	Nile tilapia (*Oreochromis niloticus*)	1% SSD	115 patients (57 FSG, 58 SSD)	11 days	Re-epithelialization time, n. of dressings, costs, pain	Reduced healing time (−0.5 days), n. of dressings, pain and costs (−42.1%) with FSG.
Lima Júnior et al., 2021 [29]	Randomized Pilot Clinical Study	Flame, SPTB	Nile tilapia (*Oreochromis niloticus*)	Sodium carboxymethylcellulose with silver (NaCMC-Ag)	24 patients	11 days	N. of dressings, pain (VAS)	Fewer dressings (1 vs. 2) and significantly lower pain (*p* = 0.0142) in the FSG group.
Shi et al., 2020 [25]	Comparative Animal Study	Flame, SPTB, DPTB	Grass carp (*Ctenopharyngodon idellus*)	Porcine collagen (PSC), gauze, Vaseline gauze	2 New Zealand rabbits	28 days	Water uptake, water vapor transmission rate	Complete healing at 28 days for collagen groups (fish and porcine). Superior water uptake for fish collagen.
Lima Júnior et al., 2020 [35]	Case Report	Flame, SPTB	Nile tilapia (*Oreochromis niloticus*)	None	1 patient (10% TBSA, SPTB)	10 days	Re-epithelialization time	Complete re-epithelialization in 10 days.
Lima Júnior et al., 2020 [30]	Phase II RCT	Flame, SPTB, DPTB	Nile tilapia (*Oreochromis niloticus*)	1% SSD	62 patients (3 study arms by depth/TBSA)	23 days	Re-epithelialization time, pain, n. of dressings	Reduced re-epithelialization time by 1.1 to 3.2 days in favor of tilapia over SSD.
Lima Júnior et al., 2020 [31]	Phase II Pilot Study	Flame, SPTB	Nile tilapia (*Oreochromis niloticus*)	1% SSD	30 pediatric patients	11 days	Re-epithelialization time, n. of dressings	Complete re-epithelialization at day 10: 86.7% (tilapia) vs. 53.3% (SSD). Significantly fewer dressings required.
Costa et al., 2019 [36]	Case Report	Flame, SPTB, DPTB	Nile tilapia (*Oreochromis niloticus*)	None	1 pediatric patient (18% TBSA, SPTB)	10 days	Re-epithelialization time	Complete re-epithelialization in 10 days.
Sandness B et al., 2019 [37]	Case Report	Flame, SPTB, DPTB	North Atlantic cod (*Gadus morhua*)	None	1 dog (10% TBSA, FTB)	19 days	Wound dimensions (length, width)	95% reduction in wound size after 56 days.
Lima Junior et al., 2019 [39]	Case Report	Combat injury, SPTB, DPTB	Nile tilapia (*Oreochromis niloticus*)	None	1 patient (16% TBSA, SPTB)	17 days	Re-epithelialization time	Complete re-epithelialization in 12 and 17 days for the two upper limbs.
Alam et al., 2019 [38]	Case Series	Flame, SPTB, DPTB	North Atlantic cod (*Gadus morhua*)	None	10 patients (STSG donor sites)	16 days	Re-epithelialization time, pain, infection	Complete re-epithelialization in an average of 11.5 days. Low pain scores. No infections.

**Table 2:** Summary of wound healing times reported with fish skin grafts according to burn depth and study model.

Study	Burn Depth	Animal/Patient	Fish Skin Graft	Wound Healing Time
Yoon et al., 2022 [27]	SPTB and DPTB	Humans	Nile tilapia (*Oreochromis niloticus)*	9.1 ± 1.0 days for group 1 treated with FSG, 10.7 ± 1.5 days for group 2 treated with FSG.
Lima et al., 2021 [28]	SPTB and DPTB	Humans	Nile tilapia (*Oreochromis niloticus*)	9.7 ± 0.6 days for complete re-epithelialization.
Lima et al., 2020 [30]	SPTB and DPTB	Humans	Nile tilapia (*Oreochromis niloticus*)	9.77 ± 0.83 days for SPTB group A, 10.56 ± 1.13 days for SPTB group B, 18.10 ± 0.99 days for DPTB group C.
Lima et al., 2019 [39]	SPTB and DPTB	Humans	Nile tilapia (*Oreochromis niloticus*)	12 days for SPTB, 17 days for DPTB.
Lima et al., 2020 [35]	SPTB	Humans	Nile tilapia (*Oreochromis niloticus*)	10 days for complete re-epithelialization.
Lima et al., 2020 [31]	SPTB	Humans	Nile tilapia (*Oreochromis niloticus*)	10.07 ± 0.46 days.
Wei et al., 2023 [22]	DPTB	Kumming mice	Silver carp (*Hypophthalmichthys molitrix*)	14 days for a wound healing rate of 93.89% ± 3.15%.
Wallner et al., 2022 [34]	DPTB	Humans	North Atlantic cod (*Gadus morhua*)	22 ± 6.3 days.
Varon et al., 2023 [23]	DPTB	Pigs	North Atlantic cod (*Gadus morhua*)	28 days for 100% re-epithelialization rate.
Stone et al., 2021 [24]	DPTB	Yorkshire pigs	North Atlantic cod (*Gadus morhua*)	28 days for a re-epithelialization of >90%.

## Data Availability

All data are contained within the article.

## Abbreviations

The following abbreviations are used in this manuscript:

FSG	Fish skin graft
DPTB	Deep partial-thickness burn
FTB	Full-thickness burn
SPTB	Superficial partial-thickness burn
STSG	Split-thickness skin graft
TBSA	Total body surface area
ADM	Acellular dermal matrix
SSD	Silver sulfadiazine
PSC	Porcine skin-derived collagen
PUFAs	Polyunsaturated fatty acids
LCUFAs	Long-chain unsaturated fatty acids
DHA	Docosahexaenoic acid
EPA	Eicosapentaenoic acid
SPMs	Specialized pro-resolving mediators
VAS	Visual analogue scale
CFU	Colony-forming units
IHS	Irradiated human skin
BTM	Biodegradable temporizing matrix
HAM	Hyaluronic acid ester matrix
PLA	Polylactic acid
POSAS	Patient and observer scar assessment scale
NaCMC-Ag	Sodium Carboxymethylcellulose impregnated with Silver
